# The influence of the rotational speed of the meat cutter knives and bowl on the microstructure of meat products

**DOI:** 10.1038/s41598-022-19566-x

**Published:** 2022-09-15

**Authors:** Mirosława Krzywdzińska-Bartkowiak, Michał Piątek, Ryszard Kowalski

**Affiliations:** Department of Meat Technology, Faculty of Food Science and Nutrition, Poznań University of Life Sciences, Wojska Polskiego 28, 60-637 Poznań, Poland

**Keywords:** Biological techniques, Optical imaging

## Abstract

The aim of the study was to determine the structure of meat batter and processed meat products, depending on the chopping time and rotational speed of the cutter knives and bowl, by means of histochemical methods combined with the computer image analysis system. Finely comminuted meat batters and processed meat products were investigated. Four variants of the rotational speed of cutter knives and bowl were applied in the experiment: 1500/10 rpm, 1500/20 rpm , 3000/10 rpm and 3000/20 rpm. The chopping process lasted 10 min. After 5, 6, 8 and 10 min of chopping samples of meat batter and processed meat products were collected for histological analyses. The microstructure of structural elements (fat globules and collagen fibres) was measured using computer image analysis. The following parameters were included in a characteristic of the images: the area, circumference, length and width of fat fields; the number of fat fields analysed; the percentage of fat fields in the field under analysis; the area, circumference, length and width of collagen fibres. The computer image analysis showed that the optimal speed of the cutter knives and bowl was 3000/20 rpm. The chopping time was reduced from 10 to 8 min.

## Introduction

Chopping is a stage in the meat production process. It consists in mechanical comminution of meat to achieve homogenous consistency and to bind all ingredients added. Chopping results in considerable comminution of the raw material, the hydration of proteins with water added during processing and the emulsification of fats. Mixing and homogenisation results in alignment of the spatial dispersion of all ingredients. These operations lead to the formation of a multi-component and multi-phase physical system known as meat batter, where the initial physical structure of all the chopped raw materials has been changed considerably. It is mostly manifested by changes in the properties of meat proteins and the fatty material^1–8^. Sodium chloride plays an important role in the extraction of myofibrillar proteins. Myofibrillar proteins are responsible for the development of functional properties of emulsified meat products, such as gel formation, water-holding capacity and emulsification^9^. The addition of fat to meat products affects their rheological and structural properties and provides a unique taste profile^8,10,11^. Fat affects the texture, flavour, mouthfeel, overall sensation of lubricity and appearance of meat products^12,13^. The structure and physicochemical properties of proteins and lipids influence the formation and stability of emulsions by forming an interfacial protein film around fat globules in finely comminuted meat products and thus they affect the texture of many food products^14–19^.

Chopping should ensure optimal fragmentation of the muscle tissue, connective tissue and adipose tissue and equal dispersion of fat at the dispersion phase. The chopping process should end when there is balance between the desirable and adverse phenomena. Undesirable phenomena result from the frictional forces of the cutting elements, i.e. the knives against the meat batter. As a result, the battering temperature rises, causing local thermal denaturation of the protein. This changes the water absorption of the meat batter and releases previously bound water. If excessive temperature rise occurs during chopping, the protein matrix may be partially denatured and broken giving rise to an unprotected fat dispersion. Increased water and fat separation will reduce both yield and quality of the final product. The chopping process should be finished at the optimal time^20,21^.

An important role in the extraction of the myofibrillar proteins plays sodium chloride Myofibrillar proteins are responsible for development of functional properties of emulsified meat products, such as the gel formation, water-holding capacity and emulsification^9^. Addition of fat to meat products plays an important role in the rheological and structural properties as well as providing a unique taste profile^8,10^. Fat contributes to the texture, flavor, mouthfeel, overall sensation of lubricity and appearance of meat products^12,13^.

For many years research institutions have been conducting investigations on meat and poultry to determine the relationship between the properties of raw materials, the basic composition of meat batter, the meat batter production technique and technology, and the quality of finished products. Due to the fact that finely comminuted meat products are commonly consumed and their quality largely depends on the raw meat batter structure it is necessary to search for quick and objective evaluation methods. One of them is computer image analysis, which is gaining popularity in the food industry. It is used to control the quality^22,23^, classify^24^ and assess a broad spectrum of raw and processed food products^25–29^. The aim of the study was to determine the influence of the rotational speed of the cutter knives and bowl on the structure of finely comminuted meat batters and processed meat products by means of histochemical methods and computer analysis of the microscope image.

## Results and discussion

### The influence of the rotational speed of the meat cutter knives and bowl and the chopping time on the comminution of fat particles in meat batters and processed meat products

**Figure 1:**
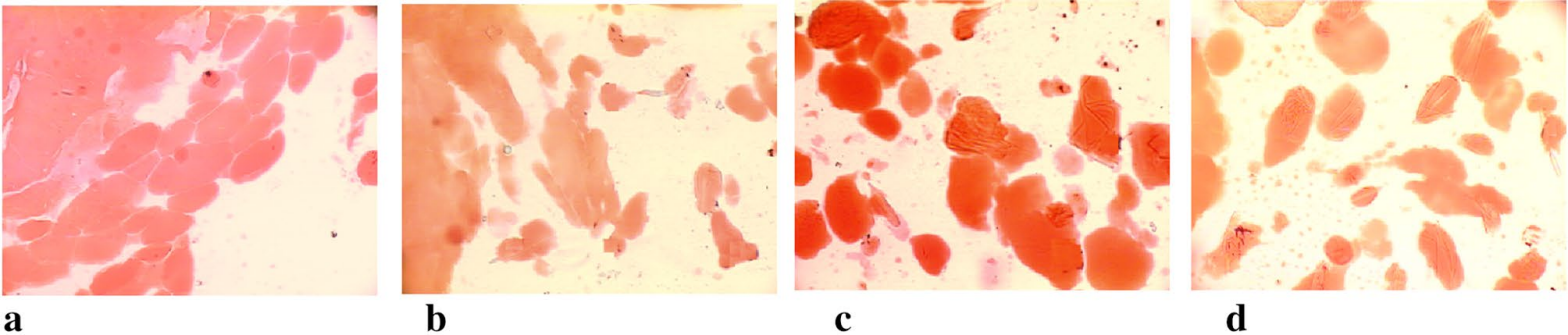
Microstructure of batter (x200) (fat globules) after 5 min chopping produced with varying rotational speed of chopper knives and bowl (**a**) −1500/10 rpm^−1^, (**b**) −1500/20 rpm^−1^, (**c**) −3000/10 rpm^−1^, (**d**) −3000/20 rpm^−1^.

**Figure 2:**
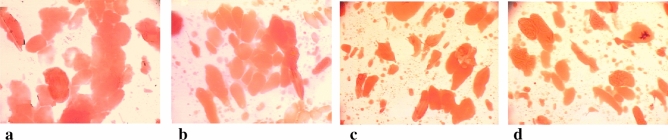
Microstructure of batter (x200) (fat globules) after 6 min chopping produced with varying rotational speed of chopper knives and bowl: (**a**) −1500/10 rpm^−1^, (**b**) −1500/20 rpm^−1^, (**c**) −3000/10 rpm^−1^, (**d**) −3000/20 rpm^−1^.

**Figure 3:**
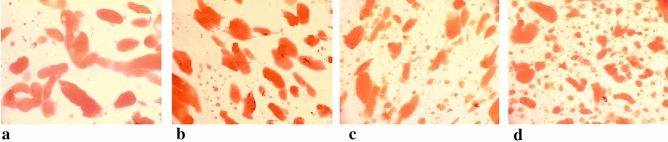
Microstructure of batter (x200) (fat globules) after 8 min chopping produced with varying rotational speed of chopper knives and bowl: (**a**) −1500/10 rpm^−1^, (**b**) −1500/20 rpm^−1^, (**c**) −3000/10 rpm^−1^, (**d**) −3000/20 rpm^−1^.

**Figure 4:**
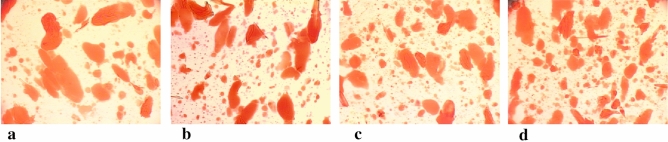
Microstructure of batter (x200) (fat globules) after 10 min chopping produced with varying rotational speed of chopper knives and bowl: (**a**) −1500/10 rpm^−1^, (**b**) −1500/20 rpm^−1^, (**c**) −3000/10 rpm^−1^, (**d**) −3000/20 rpm^−1^.

**Figure 5:**
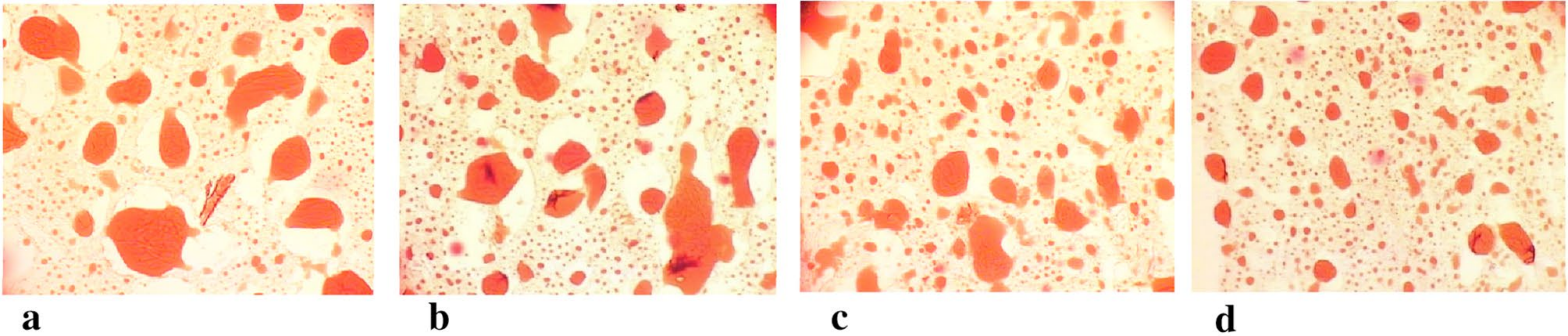
Microstructure of sausage (x200) produced with varying rotational speed of chopper knives and bowl: (**a**) −1500/10 rpm^−1^, (**b**) −1500/20 rpm^−1^, (**c**) −3000/10 rpm^−1^, (**d**) −3000/20 rpm^−1^.

The best comminution and dispersion of fat in the protein matrix was observed in the meat batter and processed meat products made at the cutter knife and bowl rotational speeds of 3000/20 rpm. The meat batters which had been chopped at these knife and bowl rotational speeds were characterised by the smallest dimensions of fat particles (Table 1), and unit area (Table 2) until 8 min of the chopping process. They also had the most fat fields (Table 3). Moreover, this may also be observed when comparing images of microstructure of produced batters, in which stained fat was presented (Figs. 1, 2, 3, 4, 5). In the meat batters, the production of which was carried out at the rotational speed of the knives of 1500 rpm, an increase in the surface of fat particles was observed, followed by its gradual decrease until the cutting process was completed.

**Table 1:** The characteristics of the geometrical parameters of fat particles vs the rotational speed of the cutter knives and bowl (1500/10 rpm, 1500/20 rpm, 3000/10 rpm, 3000/20 rpm).

Chopping time [min]	Rotational speed (rpm)		Geometrical parameters of fat particles							
			Area		Perimeter		Length		Width	
	Cutter knives	Cutter bowl	$\overline{x }$ log	$\overline{x }$[μm^2^]	$\overline{x }$ log	$\overline{x }$[μm]	$\overline{x }$ log	$\overline{x }$[μm]	$\overline{x }$ log	$\overline{x }$[μm]
5	1500	10	2.34	218.78^a^ ± 7.8	1.72	52.48^a^ ± 3.0	1.28	19.05^a^ ± 3.0	1.13	13.49^a^ ± 2.8
		20	2.70	501.19^b^ ± 5.8	1.93	85.11^b^ ± 2.6	1.50	31.62^c^ ± 2.6	1.31	20.42^b^ ± 2.6
	3000	10	2.78	602.56^bc^ ± 5.3	1.98	95.50^b^ ± 2.5	1.55	35.48^b^ ± 2.5	1.34	21.88^b^ ± 2.5
		20	2.82	660.69^c^ ± 8.1	2.00	100.00^b^ ± 3.3	1.57	37.15^b^ ± 3.2	1.35	22.39^b^ ± 3.2
6	1500	10	2.69	489.78^a^ ± 6.8	1.92	83.18^b^ ± 2.9	1.47	29.51^ab^ ± 3.0	1.31	20.42^b^ ± 2.7
		20	2.76	575.44^b^ ± 4.8	1.96	91.20^b^ ± 2.4	1.53	33.88^b^ ± 2.4	1.34	21.88^b^ ± 2.4
	3000	10	2.74	549.54^b^ ± 4.9	1.96	90.89^b^ ± 2.6	1.53	33.28^b^ ± 2.3	1.33	21.38^b^ ± 2.3
		20	2.59	389.05^a^ ± 6.8	1.86	72.44^a^ ± 3.0	1.43	26.92^a^ ± 2.9	1.25	17.78^a^ ± 2.8
8	1500	10	2.65	445.86^c^ ± 4.7	1.90	79.43^c^ ± 2.3	1.47	29.51^c^ ± 2.3	1.29	19.50^c^ ± 2.3
		20	2.65	446.68^c^ ± 5.1	1.91	81.28^c^ ± 2.5	1.48	30.20^c^ ± 2.4	1.28	19.05^c^ ± 2.5
	3000	10	2.49	309.03^b^ ± 6.8	1.81	64.57^b^ ± 3.0	1.38	23.99^b^ ± 2.9	1.18	15.14^b^ ± 2.8
		20	2.39	245.47^a^ ± 5.5	1.72	52.48^a^ ± 2.6	1.28	19.05^a^ ± 2.6	1.12	13.18^a^ ± 2.5
10	1500	10	2.46	288.40^a^ ± 6.7	1.78	60.26^a^ ± 2.9	1.35	22.39^a^ ± 2.9	1.18	15.14^a^ ± 2.7
		20	2.53	338.84^ab^ ± 6.0	1.83	67.61^b^ ± 2.8	1.40	25.12^b^ ± 2.7	1.21	16.22^ab^ ± 2.7
	3000	10	2.56	363.08^b^ ± 5.3	1.85	70.79^b^ ± 2.6	1.42	26.30^b^ ± 2.5	1.24	17.38^a^ ± 2.5
		20	2.67	467.74^c^ ± 4.1	1.91	81.28^c^ ± 2.2	1.48	30.20^c^ ± 2.2	1.30	19.95^c^ ± 2.2
Sausage	1500	10	2.01	102.33^c^ ± 3.63	1.53	33.88^a^ ± 2.04	1.09	12.30^b^ ± 2.04	0.96	9.12^a^ ± 2.0
		20	2.07	117.49^b^ ± 3.47	1.57	37.15^b^ ± 2.0	1.12	13.18^a^ ± 2.0	0.99	9.77^b^ ± 1.9
	3000	10	2.11	128.82^b^ ± 5.01	1.58	38.02^b^ ± 2.45	1.15	14.13^a^ ± 2.40	1.00	10.0^b^ ± 2.45
		20	1.97	98.33^a^ ± 2.95	1.51	32.36^a^ ± 1.86	1.06	11.48^b^ ± 1.82	0.94	8.71^a^ ± 1.82

**Table 2:** The values of the area of an individual fat particle in the meat batter and processed meat products versus the chopping time and the rotational speed of the cutter knives and bowl (1500/10, 1500/20, 3000/10 and 3000/20 rpm).

Parameter	Chopping time [min]	Rotational speed of the cutter knives [rpm]	Rotational speed of the cutter bowl [rpm]	$\overline{x }$ log	$\overline{x }$[μm^2^]
	5	1500	10	3.81	6456.54^a^ ± 1.66
			20	3.09	1230.27^b^ ± 1.2
		3000	10	3.08	1202.26^b^ ± 1.12
			20	3.06	1148.15^b^ ± 1.17
	6	1500	10	3.56	3630.78^a^ ± 1.35
			20	2.92	831.76^b^ ± 1.23
		3000	10	2.89	776.25^b^ ± 1.17
			20	2.74	588.84^c^ ± 1.20
	8	1500	10	3.31	2041.74^a^ ± 1.26
			20	2.74	851.14^b^ ± 1.20
		3000	10	2.93	1023.29^b^ ± 1.12
			20	3.01	549.54^c^ ± 1.26
	10	1500	10	2.92	831.76^a^ ± 1.23
			20	2.74	549.54^b^ ± 1.29
		3000	10	2.74	549.53^b^ ± 1.23
			20	2.76	575.44^b^ ± 1.20
The area of an individual fat particle in the processed meat products		1500	10	2.73	537.03^a^ ± 1.20
			20	2.62	416.87^bc^ ± 1.38
		3000	10	2.70	501.19^ab^ ± 1.45
			20	2.53	338.84^c^ ± 1.41

**Table 3:** The amount of fat particles and their percentage in the image field under analysis vs the chopping time and the rotational speed of the cutter knives and bowl (1500/10, 1500/20, 3000/10 and 3000/20 rpm).

Chopping time [min]	Rotational speed of the cutter knives and bowl [rpm]							
	1500/10		1500/20		3000/10		3000/20	
	Number of fat globules	Per cent content of fat globules [%]	Number of fat globules	Per cent content of fat globules [%]	Number of fat globules	Per cent content of fat globules [%]	Number of fat globules	Per cent content of fat globules [%]
5 min	28.20^a^	37.36^a^	64.65^b^	35.96^a^	79.55^c^	35.99^a^	80.85^c^	33.91^a^
6 min	33.40^a^	31.98^a^	84.25^b^	29.79^bc^	99.55^c^	29.03^c^	113.7^d^	33.55^a^
8 min	55.20^a^	29.16^a^	90.00^b^	27.67^a^	102.25^c^	32.46^b^	127.15^d^	33.52^b^
10 min	86.55^a^	28.88^a^	128.1^b^	30.92^ab^	141.5^b^	31.68^ab^	148.00^c^	33.58^b^
Sausage	125.6^a^	25.68^a^	212.2 b	25.87^a^	195.4 ^b^	23.89^b^	199.7 ^b^	20.20^c^

The means in the rows which are denoted by the letters a, b, c and d differ statistically significantly at *p* ≤ 0.05.

During the chopping process the percentage of fat particles did not change significantly (Table 3). As the chopping time was increasing, the area of fat particles in the meat batters was decreasing at all the knife and bowl rotational speeds until 8 min of the chopping process.

The linear correlations between the dimensions of fat particles in the meat batters under study (area, perimeter, length, width) and the chopping time and the rotational speed of the cutter knives and bowl showed that only the chopping time was statistically significant and negatively correlated with the dimensions of fat cells. The other correlations were insignificant (Table 4).

**Table 4:** The linear correlation between the parameters of the dimensions of fat particles in the meat batter and the rotational speed of the cutter knives and bowl.

Parameters of the dimensions of fat particles	Chopping time	Rotational speed of the cutter knives	Rotational speed of the cutter bowl
Area	− 0.3647 (0.040)*	0.1076 (0.558)*	− 0.1223 (0.505)*
Length	− 0.3566 (0.045)*	0.1444 (0.430)*	− 0.1083 (0.555)*
Width	− 0.5451 (0.001)*	0.0631 (0.430)*	− 0.0552 (0.764)*
Perimeter	− 0.3731 (0.035)*	0.1345 (0.463)*	− 0.1138 (0.535)*

*significance levels of correlation coefficients (*p* ≤ 0.05).

The results obtained after 10 min of chopping the meat batter produced at the knife and bowl rotational speeds of 3000/20 rpm deserve greater attention. After this chopping time there were larger values of the fat particle area than after 8 min of the process (Table 1, Fig. 5). The unit surface area of the fat particle in the meat batter after 10 min of chopping was also similar to the area measured at the knife rotational speed of 1500 rpm. The unit surface area in the processed products made from the meat batter at the speeds of 3000/20 rpm was reduced by almost half of the value noted for the meat batter (Table 2). The situation could be explained by the fact that at the cutter knife and bowl speeds of 3000/20 rpm the chopping time was too long and the fat particles which had already been fragmented began to aggregate. At higher rotational speeds the cutter knife makes more cuts in one minute and thus meat batter becomes more fragmented. We can conclude that it is possible to achieve the desirable effect in a shorter time.

### The influence of the rotational speed of the meat cutter knives and bowl and the chopping time on the unit area of fat particles (IOA).

The area occupied by fat particles was divided by their number. As a result, the value of the area occupied by an individual object was calculated. After logarithmic transformation the value was subjected to the analysis of variance. The meat batter produced at the knife and bowl rotational speeds of 1500/10 rpm was characterised by the largest average surface area of an individual fat particle. The smallest average surface area of an individual fat particle was noted at the speeds of 3000/20 rpm (Table 2).

Table 5 shows the coefficients of linear correlations between the area of individual objects and the variability factors under analysis (the chopping time, the rotational speed of the meat cutter knives and bowl). The linear regression analysis (1) showed that only the chopping time had statistically significant influence on the surface of an individual object. A multiple linear regression analysis was conducted so as to better describe the phenomenon.

1 $$\begin{aligned} & {\text{IOA}} = {4}.{11}{-}0.0{718}^*\left( {\text{T}} \right){-}0.000{1}^*\left( {{\text{KR}}} \right){-}0.0{145}^*\left({{\text{BR}}} \right);\;\;\;\;\;{\text{R}}^{{2}} = 0.{4826}; \;\;\;\;P = 0.000{3} \\ & {\text{Beta}} =\quad \;\left( { - 0.{5372}} \right)\;\;\quad \;\left( { - 0.{3381}} \right)\quad\quad\;\left( { - 0.{2822}} \right) \\ \end{aligned}$$

**Table 5:** The linear correlation between the area of individual objects and the variation factors under analysis (meat batters).

Variation factor	Individual object area
Chopping time	− 0.5372 (0.002)*
Rotational speed of the meat cutter knives	− 0.3382 (0.058)*
Rotational speed of the meat cutter bowl	− 0.2822 (0.118)*

*significance levels of correlation coefficients (*p* ≤ 0.05).

As can be seen in the multiple linear regression equation above, the chopping time had the greatest influence on changes in the surface area of an individual object (IOA) (fat particle) as a function of the chopping time (T) and the rotational speed of the meat cutter knives (KR) and bowl (BR). The rotations of the meat cutter knives and bowl had lesser influence and their influence was less diversified.

The processed meat products made from the meat batter chopped at the rotational speeds of 1500/10 rpm were characterised by the largest surface area of an individual fat particle. The smallest surface area of an individual fat particle was noted in the processed products made from the meat batter chopped at the rotational speeds of 3000/20 rpm. The values of this parameter were significantly different from the values noted in the other variants. The calculations of linear correlations between the area of an individual fat particle and the influence of the rotation of the cutter knives and bowl showed that the rotation of the bowl had statistically significant influence on this parameter (Table 6).

**Table 6:** The linear correlation between the area of individual objects and the variation factors under analysis (the processed meat products).

Variation factor	Individual object area
Rotational speed of the meat cutter knives	− 0.1866 (0.097)*
Rotational speed of the meat cutter bowl	− 0.4427 (0.000)*

*significance levels of correlation coefficients (*p* ≤ 0.05).

The multiple linear regression Eq. (2) as a function of the rotations of the meat cutter knives and bowl showed that at the set chopping time the bowl rotations had greater influence on the experimental meat than the knife rotations. Due to the number of degrees of freedom of the system even such a weak correlation was statistically highly significant.

2 $$\begin{aligned}& {\text{IOA}} = 2.95 \, {-}3.97{\text{E}}{-}5^*\left( {{\text{KR}}} \right) \, {-}0.0141^*\left( {{\text{BR}}} \right); \,\,\,\,{\text{R}}^{2} = 0.2308, \,\,P = 0.000; \hfill \\ & {\text{Beta}} = \quad\;\left( { - 0.1866} \right)\quad\quad\quad\quad\left( { - 0.4427} \right) \hfill \\ \end{aligned}$$

### The influence of the rotational speed of the cutter knives and bowl and the chopping time on the percentage of fat particles (PFP) and their amount in the field of view under investigation

Table 3 shows the values of the area occupied by fat particles in the field of the image under study according to the chopping time. The analysis of the data shows that after 5 min of chopping the area occupied by fat particles in the meat batter produced at the knife and bowl rotational speeds of 1500/10, 1500/20 and 3000/10 rpm was significantly reduced (Fig. 1). At the highest rotational speed of 3000/20 rpm the area occupied by fat particles remained at the same level during the whole chopping period (between 5 and 10 min).

The values of the area occupied by fat particles in the sausage for individual variants indicate that the impact of the knife and bowl rotational on the surface of the image occupied by the fat particles in the first experimental series is not unambiguous. In the second experimental series the area of the image occupied by fat particles in the processed products made from the meat batter chopped at the knife and bowl speeds of 3000/20 rpm was significantly reduced (Table 3, Fig. 5). It seems that other factors played an important role in the experiment, including the properties of the raw material.

The relations between the determinants under study can be better described with the following linear correlation (3).

3 $$\begin{aligned} &{\text{PFP }} = {32}.{123 }{-} \, 0.00{2}^*\,{\text{KR }}{-} \, 0.{175}^*\,{\text{BR}};\,\, {\text{R}}^{{2}} = 0.{218}0; \hfill \\ &{\text{Beta}} = \quad\quad\;\;( - 0.{4225})\quad\;\;\left( { - 0.{1987}} \right) \hfill \\ \end{aligned}$$

The beta coefficients indicate that the cutter knife rotations had greater influence than the bowl rotations on the percentage of fat particles in the field under analysis.

The amounts of fat particles in the meat batters increased along with the chopping time in all the variants of the cutter knife and bowl rotational speeds (1500/10 rpm, 1500/20 rpm, 3000/10 rpm and 3000/20 rpm). The comparison of the same chopping times in individual variants of the cutter knife and bowl rotational speeds showed that the amount of fat particles increased along with the rotational speed of the cutter knives and bowl. The largest amount of fat particles was found in the meat batter and processed products made at the cutter knife and bowl rotational speeds of 3000/20 rpm.

### The influence of the cutter knife and bowl rotational speeds on the dimensions of collagen fibres

The meat batter made at the knife rotational speed of 1500 rpm was characterised by the largest area, perimeter, length and width of collagen fibres (Table 7). However, it is noteworthy that these values were not always statistically significant. On the other hand, the meat batter made at the knife and bowl rotational speeds of 3000/20 rpm was characterised by the lowest values of these determinants.

**Table 7:** The dimensions of collagen fibres versus the rotational speed of the cutter knives (KR) and bowl (BR) (1500/10, 1500/20, 3000/10 and 3000/20 rpm).

Chopping time [min]	(KR) [rpm]	(BR) [rpm]	Geometrical parameters of collagen fibres							
			Area		Perimeter		Length		Width	
			$\overline{x }$ log	$\overline{x }$[μm^2^]	$\overline{x }$ log	$\overline{x }$[μm]	$\overline{x }$ log	$\overline{x }$[μm]	$\overline{x }$ log	$\overline{x }$[μm]
5	1500	10	4.01	10,232.93^a^ ± 3.3	2.79	616.60^a^ ± 2.0	2.37	234.42^a^ ± 2.0	1.92	83.18^a^ ± 1.9
		20	3.80	6309.57^b^ ± 3.3	2.65	446.68^b^ ± 1.9	2.23	169.82^b^ ± 1.9	1.81	64.57^b^ ± 1.9
	3000	10	3.60	3981.07^bc^ ± 3.8	2.55	354.81^bc^ ± 2.0	2.12	131.83^b^ ± 2.1	1.71	51.29^bc^ ± 2.0
		20	3.54	3467.37^c^ ± 2.7	2.52	331.13^b^ ± 1.7	2.13	134.90^b^ ± 1.7	1.61	40.74^c^ ± 1.7
6	1500	10	3.80	6309.57^a^ ± 3.4	2.68	478.63^a^ ± 1.8	2.27	186.21^a^ ± 1.9	1.77	58.88^a^ ± 2.0
		20	3.64	4365.16^ab^ ± 2.9	2.61	407.38^ab^ ± 1.7	2.18	151.36^ab^ ± 1.8	1.71	51.29^ab^ ± 1.8
	3000	10	3.56	3630.78^bc^ ± 2.6	2.53	338.84^bc^ ± 1.7	2.13	134.90^b^ ± 1.8	1.62	41.69^bc^ ± 1.6
		20	3.44	2754.23^c^ ± 2.3	2.50	316.23^c^ ± 1.6	2.10	125.89^b^ ± 1.7	1.57	37.15^c^ ± 1.6
8	1500	10	3.78	6025.60^a^ ± 2.82	2.68	478.63^a^ ± 1.78	2.26	181.97^a^ ± 1.91	1.78	60.26^a^ ± 1.7
		20	3.50	3162.28^b^ ± 2.29	2.51	323.59^b^ ± 1.66	2.08	120.23^b^ ± 1.82	1.65	44.67^b^ ± 1.55
	3000	10	3.56	3630.78^b^ ± 2.51	2.55	354.8^b^ ± 1.70	2.15	141.25^b^ ± 1.82	1.63	42.66^b^ ± 1.51
		20	3.28	2187.76^c^ ± 2.4	2.46	288.40^b^ ± 1.7	2.06	114.82^b^ ± 1.7	1.50	31.62^c^ ± 1.6
10	1500	10	3.64	4365.16^a^ ± 3.4	2.59	389.05^a^ ± 1.9	2.18	151.36^a^ ± 1.9	1.69	48.98^a^ ± 1.9
		20	3.45	2818.38^b^ ± 2.8	2.48	302.00^b^ ± 1.8	2.05	112.20^b^ ± 1.9	1.62	41.69^ab^ ± 1.7
	3000	10	3.38	2398.83^b^ ± 2.8	2.45	281.84^b^ ± 1.7	2.04	109.65^b^ ± 1.8	1.58	38.02^bc^ ± 1.8
		20	3.34	1905.46^b^ ± 2.24	2.39	245.47^c^ ± 1.55	1.97	93.33^c^ ± 1.62	1.50	31.62^c^ ± 1.58
Sausage	1500	10	3.67	4677.35^a^ ± 2.8	2.62	416.87^a^ ± 1.7	2.21	162.18^a^ ± 1.9	1.79	61.66^c^ ± 1.6
		20	3.74	4495.41^a^ ± 2.4	2.57	371.54^a^ ± 1.6	2.14	138.05^b^ ± 1.7	1.77	58.88^b^ ± 1.9
	3000	10	3.48	3019.95^b^ ± 1.8	2.50	316.23^b^ ± 1.4	2.09	123.03^b^ ± 1.5	1.62	41.69^a^ ± 1.7
		20	3.31	2041.74^c^ ± 2.1	2.42	263.03^c^ ± 1.5	2.01	102.33^c^ ± 1.6	1.51	32.36^a^ ± 1.6

The linear correlation between the parameters of collagen fibre dimensions in the meat batters, the chopping time and the rotational speed of the cutter knives and bowl (see Table 8), showed that both the chopping time and the rotational speed of the cutter knives had decisive influence on the dimensions of collagen fibres.

**Table 8:** The linear correlation between the parameters of the collagen fibre dimensions and the rotational speed of the cutter knives and bowl.

Parameters	Meat batter			Sausage	
	Chopping time	Rotational speed of the cutter knives	Rotational speed of the cutter bowl	Rotational speed of the cutter knives	Rotational speed of the cutter bowl
Area	− 0.5917 (0.000)*	− 0.5053 (0.003)*	− 0.2584 (0.153)*	− 0.3837 (0.000)*	− 0.0613 (0.221)*
Perimeter	− 0.5659 (0.001)*	− 0.5205 (0.002)*	− 0.2802 (0.120)*	− 0.3255 (0.000)*	− 0.1496 (0.003)*
Length	− 0.5844 (0.000)*	− 0.4646 (0.007)*	− 0.2822 (0.118)*	− 0.2740 (0.000)*	− 0.1616 (0.001)*
Width	− 0.5495 (0.001)*	− 0.5827 (0.000)*	− 0.2889 (0.109)*	− 0.4285 (0.000)*	0.0137 (0.785)*

*significance levels of correlation coefficients (*p* ≤ 0.05).

These dependencies can be described with the following regression equations:

4 $$\begin{aligned} &{\text{Area}} = {4}.{42 }{-}{1}.{\text{46E}}{-}0{4}^*\,{\text{rpm }}{-} \, 0.0{67}^*\, {\text{min}};\,\, {\text{R}}^{{2}} =0.{6}0{55},\,\, {{p}} < 0.000; \hfill \\ &{\text{Beta}} = \quad\;\;\left( { - 0.{5}0{53}} \right)\qquad\quad\,\,\,\left( { - 0.{5917}} \right) \hfill \\ \end{aligned}$$

5 $$\begin{aligned} &{\text{Perimeter}} = {2}.{97 }{-}{7}.{\text{58E}}{-}0{5}^*{\text{ rpm }}{-} \, 0.0{32}^*\, {\text{min}}; \,\,\,{\text{R}}^{{2}} =0.{591}0, \,\,{{p}} < 0.000; \hfill \\ &{\text{Beta}} = \quad\qquad\;\;\left( { - 0.{52}0{4}} \right)\quad\quad\quad\;\,\,\;\;\left( { - 0.{5658}} \right) \hfill \\ \end{aligned}$$

6 $$\begin{aligned} & {\text{Length}} = {2}.{54 }{-}{6}.{\text{79E}}{-}0{5}^*{\text{ rpm}}{-} \, 0.0{33}^*\, {\text{min}};\;\;\;\; {\text{R}}^{{2}} =0.{5573},\,\; {{p}} < 0.000; \\ & {\text{Beta}} = \quad \quad\;\;  \left( { - 0.{4645}} \right)\;\quad \qquad\;\;\left( { - 0.{5843}} \right) \\ \end{aligned}$$

7 $$\begin{aligned} &{\text{Width}} = {2}.{16}-{9}.{\text{91E}}{-}0{5}^*{\text{ rpm }}{-} \, 0.0{36}^*\,{\text{min}};\,\,\, {\text{R}}^{{2}} =0.{6414}, \,\,\,{{p}} < 0.000; \hfill \\ &{\text{Beta}} = \quad\quad\;\;\left( { - 0.{5826}} \right)\quad\quad\quad\;\;\;\;\left( { - 0.{5494}} \right) \hfill \\ \end{aligned}$$

As can be concluded from the multiple linear regression equations, the dimensions of collagen fibres decreased as the rotational speed of the cutter knives and the chopping time increased. The chopping time and the rotational speed of the cutter knives had roughly equal influence on the comminution of collagen fibres as the values of beta coefficients were very similar.

The processed products made from the meat batter chopped at the knife and bowl rotational speeds of 3000/20 rpm was characterised by the smallest dimensions of collagen fibres, and their values were statistically significantly different from those noted in the other variants (Table 7). The linear correlation between the parameters of collagen fibres and the rotational speed of the cutter knives and bowl (Table 8) showed that all the parameters of collagen fibres were statistically significantly and negatively correlated with the rotational speed of the cutter knives. There was a much weaker though significant dependency between the rotational speed of the bowl and the perimeter and length of collagen fibres.

The high comminution of collagen fibres after 10 min of chopping may indicate that the process was too long. According to Haack et al.^30^, in order to achieve the desired degree of comminution meat should be chopped for a shorter period of time at high rotational speeds, because the sharpness of the knife deteriorates as its working time increases and the degree of equal comminution is reduced. Apart from that, the state of emulsification is exceeded, the temperature of the meat batter rises and its quality decreases. Therefore, it is necessary to use higher durability knives and reduce the load by processing small pieces of raw material. This can be achieved by initial comminution of meat with a mincer into smaller particles than 3 mm. There were similar results of the experiment conducted by Curt et al.^31^, who tested the Response Surface Methodology to optimise the comminution conditions in the cutter, using knife rotational speeds of 500–3500 rpm and the chopping time up to 6 min. The researchers tested 5 parameters: the size of fat particles, homogenisation, cohesion, hardness and binding. The highest quality meat batter was obtained when meat was chopped with knives rotating at speeds of 2000–3500 rpm for 3–5 min. The chopping process was the most economical when the knives rotated at a speed of 2000 rpm for 3 min.

## Materials and methods

### Research material

The research was conducted on meat batters and finely comminuted meat products made in four variants of the rotational speed of the cutter knives and bowl: 1500/10 rpm, 1500/20 rpm, 3000/10 rpm and 3000/20 rpm. Hind ham hock muscles and fine ham fat were used as raw materials for the production of finely comminuted meat batters and processed meat products. The raw materials were collected directly from meat processing plants. The model product recipe looked as follows: 70% of ham hock pork with tendons, 30% of fine ham fat, 40% of water with ice added at an adequate proportion to the fat and meat mass and 2.0% of curing salt.

During the chopping process, after the same period of time samples were collected from all the meat batter variants to prepare specimens for histological analysis.

### Cutter technical specifications

The finely comminuted meat batters were produced in a two-speed cutter, where the rotational speeds of the cutter knives were 1500 rpm and 3000 rpm, whereas the rotational speeds of the bowl were 10 rpm and 20 rpm. The cutter bowl capacity was 22 dm^3^. There were four broken-line-shaped knives mounted on the knife shaft.

### Technological process

The meat and fat raw materials for the production of model meat batter were crushed in a mincer, through a 3-mm-hole net. A curing mix was added to the meat and it was cured for 24 h at 4–6℃. Next, the raw materials were chopped into a bowl in the following order: meat, ice with water and fat. The chopping process lasted 10 min. The final temperature of the meat batters did not exceed 12℃. The meat batters were placed in natural casings with a diameter of 28–30 mm. Next, the processed meat products were dried at 35℃ for 30 min, smoked at 60℃ and scalded at 75℃ in a smoking-scalding chamber until the temperature in the geometrical centre of the bar was 70℃. Then, the processed meat products were cooled in cold water and after 24 h of cold storage at 4–6℃ they were analysed.

### Preparation of histological specimens

Samples of finely comminuted meat batters were collected after 5, 6, 8 and 10 min of chopping and from finished products to prepare specimens for histological analysis.

Blocks sized 10 × 10x10 mm were made from the meat batter and processed meat samples and they were frozen in liquid nitrogen. Next, the blocks were transferred to a cryostat and sheared into 10 μm scraps The scraps were placed on protein-coated primary slides and dried at room temperature for about 30 min. Then the specimens were stained with Oil Red O to show the fat dispersion. Van Gieson's stain was applied to observe changes in the connective tissue, mainly collagen^32^.

### Computer image analysis

The histological specimens were subjected to computer image analysis. The image from an Axiolab microscope was transmitted by a camera to a computer, where it was analysed with the MultiScan software v.13.01. An identical procedure of object identification and analysis was prepared for all the specimens. The structure of the specimens was examined at a constant magnification of the microscope (× 200). 10 fields of a constant surface area were analysed in each specimen. The images were characterised according to the following parameters: the area, length, width and circumference of fat fields; the number of fat fields analysed; the percentage of fat fields in the field under analysis; the area, length, width and circumference of collagen fibres. Additionally, the area of an individual fat particle was calculated by dividing the total area occupied by fat in the field under analysis by the number of fat particles^33–35^.

### Statistical analysis

As there was a very wide range of values of the numerical data obtained with computer image analysis, they were transformed into the following form: Y = log (x). The Kolmogorov–Smirnov test revealed that the logarithmic transformation resulted in a normal distribution of data. This procedure was recommended by Wagner and Błaczak^36^. The diversification of mean values was assessed with the t-test. The results of analyses were presented in two forms: as logarithms and as true values. In this case the standard deviation was not a measure of precision of the calculations due to the fact that the results of standard deviations are linear for small logarithmic values, but for larger values they have exponential nature. The statistical significance of the effect of the factors was assessed using two-way analysis of variance (ANOVA) at a significance level of *p* ≤ 0.05. It was assumed that the effect of the chopping time on the parameters was obvious. This assumption is consistent with the data reported by Elandt^37^ and Karpiski^38^.

## Conclusion

The results of the research based on measurements of the dimensions of fat particles and collagen fibres in finely comminuted meat batters and the resulting processed products by means of the computer image analysis system confirmed the possibility to use this method to assess the quality of meat and fat emulsion and the end product made from it. The images of the microstructure of the meat batters and processed meat products obtained during the study enabled the identification of the objects under analysis (fat particles and collagen fibres). The MultiScan program enabled the characterisation of variation in their main geometrical parameters. The computer image analysis showed that the rotational speed of the cutter knives and bowl had statistically significant influence on the comminution of meat batters. Apart from that, it also allowed us to determine the optimal rotational speed of the cutter knives and bowl, i.e. 3000/20 rpm.

It was found that the fact that at the cutter knife and bowl speeds of 3000/20 rpm the chopping time 10 min was too long and the fat particles which had already been fragmented began to aggregate. At higher rotational speeds the cutter knife makes more cuts in one minute and thus meat batter becomes more fragmented. We can conclude that it is possible to achieve the desirable effect in a shorter time. The results of the research and the data presented in reference publications allowed us to use the optimal rotational speed of the cutter knives and bowl, i.e. 3000/20 rpm in further experiments and shorten the chopping process from 10 to 8 min.
